# Relative Contribution of Dengue IgG Antibodies Acquired during Gestation or Breastfeeding in Mediating Dengue Disease Enhancement and Protection in Type I Interferon Receptor-Deficient Mice

**DOI:** 10.1371/journal.pntd.0004805

**Published:** 2016-06-24

**Authors:** Pei Xuan Lee, Li Ching Ong, Eshele Anak Libau, Sylvie Alonso

**Affiliations:** 1 Department of Microbiology and Immunology, Yong Loo Lin School of Medicine, National University of Singapore, Singapore; 2 Immunology Programme, Life Sciences Institute, National University of Singapore, Singapore; University of California, Berkeley, UNITED STATES

## Abstract

Dengue virus (DENV) causes a spectrum of diseases ranging from self-limiting dengue fever to severe conditions such as haemorrhagic fever and dengue shock syndrome. Antibody-dependent enhancement (ADE) is thought to explain the occurrence of severe dengue whereby pre-existing binding but non-neutralising antibodies enhance DENV infection. The ADE phenomenon is supported by epidemiological findings that infants that born to dengue immune mothers are at greater risk to develop severe dengue upon primary infection. The role of maternally acquired dengue-specific antibodies in disease enhancement was recently recapitulated in a mouse model where mice born to DENV1-immune mothers experienced enhanced disease severity upon DENV2 infection. Here, this study investigates the relative contribution of maternal dengue-specific antibodies acquired during gestation and breastfeeding in dengue disease. Using a surrogate breastfeeding mother experimental approach, we showed that majority of the maternal dengue-specific antibodies were acquired during breastfeeding and conferred an extended enhancement window. On the other hand, in the context of homologous infection, breastfeeding conferred protection. Furthermore, measurement of dengue-specific antibody titres over time in mice born to dengue immune mothers revealed a biphasic pattern of antibody decay as reported in humans. Our work provides evidence of the potential contribution of breast milk-acquired dengue-specific IgG antibodies in enhancement and protection against dengue. Should such contribution be established in humans as well, it may have important implications for the development of guidelines to dengue-immune breastfeeding mothers.

## Author Summary

Epidemiological observations showed that 5–9 month old infants born to dengue immune mothers have increased risk of developing severe disease upon primary dengue infection. This disease enhancement has been associated with the presence of binding but non-neutralizing maternal dengue antibodies. The recent development of experimental dengue mouse models involving maternal antibodies supports their role in both disease enhancement and protection. Here, we examined the contribution of maternal antibodies acquired during gestation and breastfeeding in disease enhancement and protection. Our findings support that majority of maternal IgG antibodies circulating in mice born to dengue immune mothers are acquired from breast milk. As such, we showed that breastfeeding conferred extended window of enhancement or protection. These findings provide the first experimental evidence for a role of breast milk dengue antibodies in mediating dengue infection outcome. This may help develop guidelines to dengue immune breastfeeding mothers.

## Introduction

Dengue is a mosquito-borne viral disease responsible for an estimated 390 million annual dengue infections in the tropical and sub-tropical regions [1]. While most infected individuals manifest as asymptomatic or self-limiting dengue fever (DF), a significant proportion progresses to more severe conditions—dengue haemorrhagic fever (DHF) and dengue shock syndrome (DSS)–characterised by symptoms such as vascular leakage and haemorrhage, which could be fatal [2, 3]. The lack of effective vaccine and treatment against the potentially life-threatening dengue poses a serious public health concern.

Dengue virus (DENV), the etiological agent responsible for dengue, consists of four distinct serotypes (2). Infection with one serotype confers long-term protection against the same serotype but only short-term protection against the other serotypes [4]. On the other hand, antibodies generated during primary infection may cause enhancement of dengue disease, a phenomenon coined as antibody-dependent enhancement (ADE) [3, 5–8]. ADE develops due to the presence of pre-existing sub-neutralising antibodies that opsonise but do not effectively neutralise the virus. This results in the binding and endocytosis of virus-antibody immune complexes to Fcγ receptors (FcγR)-bearing cells such as monocytes and macrophages. However, instead of being degraded within the endosome, the virus escapes and replicates within the cells, thereby making the FcγR-mediated virus entry an efficient way to produce virus progeny. Furthermore, the antibody-mediated internalisation of DENV was shown to suppress innate antiviral responses, which further enhanced viral production [9]. This ADE hypothesis may explain the occurrence of DHF/DSS in secondary heterotypic infections as well as in primary infections of infants who passively acquired homologous or heterologous maternal antibodies [5, 7]. Whereas the role in disease severity of other immune cells such as T cells during a secondary heterotypic infection remains a matter of debate, such possibility is clearly excluded in primary infections of infants born to dengue immune mothers, which exclusively relies on the maternal antibodies. This scenario was recently reproduced in two mouse models whereby young mice born to DENV1-immune mothers experienced enhancement of disease severity upon DENV2 infection [10, 11].

Transfer of maternal antibodies transplacentally and via breastfeeding has been known to help protect infants against pathogens during early life [12, 13]. Infants born to mothers immunised during pregnancy were protected against respective infections [14–16], indicating the role of transplacentally acquired pathogen-specific IgG antibodies in protection. On the other hand, it has been well established that breastfeeding provides IgA-mediated mucosal immunity and was shown to protect infants against pathogen-associated diarrhoea [17–20]. While protection afforded by transplacentally acquired IgG antibodies has been well studied, information on the role of IgG antibodies acquired from breastfeeding is limited [21]. Neonatal Fc receptor (FcRn) mediates maternal IgG transfer across the placenta and small intestines [22–25], allowing transfer into the circulation of IgG antibodies during gestation and breastfeeding respectively.

Here, using A129 mice (type I interferon-deficient), we studied the relative contribution of dengue-specific IgG antibodies that were acquired transplacentally or through breastfeeding in mediating dengue disease enhancement and protection. We report that in this mouse model breastfeeding represents the main route of maternal IgG transfer and that dengue antibodies present in breast milk play a critical role in enhancement or protection against dengue infection.

## Materials and Methods

### Ethics statement

All the animal experiments were carried out in accordance with the guidelines of the National Advisory Committee for laboratory Animal Research (NACLAR). Animal facilities are licensed by the regulatory body Agri-Food and Veterinary Authority of Singapore (AVA). The described animal experiments were approved by the Institutional Animal Care and Use Committee (IACUC) from National University of Singapore (NUS) under the protocol number R14-97.

### Virus strains and growth conditions

DENV1 (Dengue 1 05K3903DK1) was isolated during dengue outbreak in Singapore in 2005. DENV2 (Dengue 2 D2Y98P-PP1) was derived from a clinical strain isolated in Singapore in 2000. All DENV stocks were generated in C6/36 *Aedes albopictus* cell line (American Type Culture Collection (ATCC) #CRL-1660), maintained in Leibovitz’s L-15 medium (GIBCO) supplemented with 2% fetal bovine serum (FBS) as previously described [10]. Viruses were stored at -80°C. Virus titres were determined via plaque assay in BHK-21 cells (See section below). Concentrated virus stocks were also prepared by precipitating viruses with 14% (w/v) polyethylene glycol (PEG) and subsequently used for IgG antibody quantification.

### Virus quantification

Plaque assay was performed using BHK-21 cells (ATCC #CCL-10) as previously described (10). BHK-21 cells were cultured in 24-well plates (NUNC). Cells monolayers reaching 80% confluency were infected with serially diluted viral suspensions. After 1 hour incubation at 37°C, 1% (w/v) carboxymethyl cellulose-containing RPMI-1640 medium supplemented with 2% FBS was added. After incubation for 4 (DENV2) or 5 (DENV1) days, cells were fixed with 4% paraformaldehyde and stained with 1% crystal violet. Plaques were counted and expressed as the number of plaque forming unit per mililiter (PFU/mL).

### ADE infection mouse model and switching conditions

**Fig 1:**
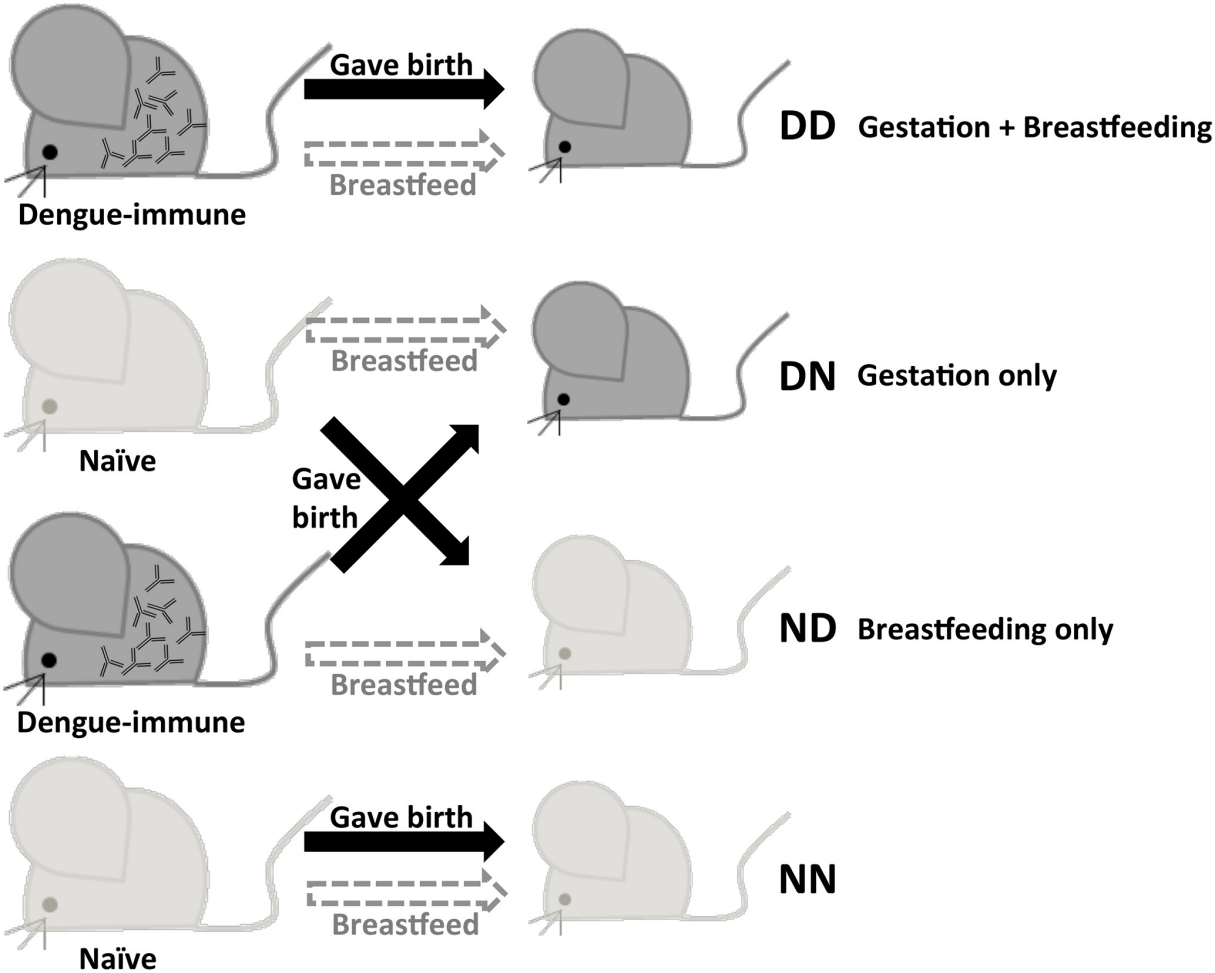
The breastfeeding surrogate mother experiment. Adult female A129 mice were iv. infected with 10^6^ PFU per mouse of DENV1. One week post-infection after virus clearance, the females were mated with naïve adult males. Age-matched naïve females were also mated concurrently. At birth, pups born to either DENV1-immune or naïve mothers were switched and nursed by naïve or dengue-immune mothers respectively. Control groups comprised of mice nursed by birth mothers were also included. DD: Born to and nursed by dengue-immune mother; DN: Born to dengue-immune mother, but nursed by naïve mother; ND: Born to naïve mother, but nursed by dengue-immune mother; NN: Born to and nursed by naïve mother.

Adult A129 females were infected with 10^6^ PFU per mouse of DENV1 or DENV2 via intravenous (iv) route. One week post-infection after virus clearance, the females were mated with naïve adult males. Age-matched naïve females were also mated concurrently. At birth, pups born to either dengue-immune or naïve mothers were switched and nursed by naïve or dengue-immune mothers respectively (Fig 1). Control groups comprised of mice nursed by birth mothers were also included. All pups were breastfed by the respective mothers and were weaned out 21 days later. At 5 and 10 weeks of age, these mice were challenged with either 10^6^ PFU (sub-lethal dose) or 10^7^ PFU (lethal dose) of DENV2 via iv route and monitored for survival and bled at specific time point for viremia determination. The infected animals were monitored daily for clinical manifestations. The scoring system used was: 0: Healthy; 1: Ruffled fur; 2: Hunched back; 3: Diarrhoea; 4: Lethargic; 5: Moribund. Survival rate was derived from the number of mice that were euthanized at moribund stage as evidenced by severe diarrhoea and extreme lethargy as described previously [11]. Serum collection was performed at various time points after birth for antibody measurement, or at day 4 post-infection to measure virus titres by plaque assay.

### Measurement of DENV-specific total IgG and IgA titre

Levels of systemic IgG and IgA antibodies specific to DENV1 or DENV2 were quantified via indirect enzyme-linked immunosorbent assay (ELISA). UV-inactivated viruses (150 ng) were coated onto 96-well ELISA plates (Corning costar) and incubated at 4°C overnight. Serially diluted serum samples were added to wells and incubated at 37°C for 1 hour. HRP-labelled goat anti-mouse IgG (H+L) (Bio-rad) or anti-mouse IgA (Thermo-Scientific) was subsequently added and incubated at 37°C for 1 hour. Detection was done with the addition of *o*-phenylenediamine dihydrochloride substrate SigmaFast (Sigma Aldrich). The reaction was stopped upon addition of 1 M H_2_SO_4_. Absorbance was read at 490 nm, and titre was calculated by nonlinear regression as the reciprocal of the highest serum dilution with absorbance corresponding to 3 times the blank absorbance.

### Plaque reduction neutralisation test (PRNT)

Heat-inactivated serum samples were serially diluted with RPMI-1640 medium supplemented with 2% FBS containing approximately 500 PFU/mL DENV1 or DENV2. After 1 hour incubation at 37°C, plaque assay was performed in BHK cells. The percentage neutralization at each dilution was calculated based on the reduction in plaque number compared to that of the positive control. PRNT_50_ was determined by nonlinear regression as the reciprocal of the highest serum dilution that gave 50% reduction in number of plaques.

### Statistical analysis

Data were analysed using Mann-Whitney statistical test using Graphpad Prism. Difference were considered significant (*) at *p* value <0.05.

## Results

### Maternal dengue-specific antibodies are mainly acquired from breastfeeding

In a previous work, we showed that 5-week old A129 mice born to DENV1-immune mothers displayed enhancement of disease severity upon heterologous DENV2 infection [11]. To investigate the relative contribution of dengue-specific IgG antibodies acquired during gestation and breastfeeding in disease enhancement, mice born to DENV1-immune were switched at birth and nursed by surrogate breastfeeding dengue naïve mothers (Fig 1). Similarly, mice born to naïve dams were nursed by DENV1-immune mothers. Thus, DENV1 specific-IgG antibodies circulating in mice born to DENV1-immune mothers but nursed by naïve mother (DN) were only acquired during gestation. On the other hand, DENV1-IgG antibodies circulating in mice born to naïve mother but nursed by DENV1-immune mother (ND) were only acquired during breastfeeding. Control groups included mice born to and nursed by DENV1-immune mothers (DD), and mice born to and nursed by naïve mothers (NN).

**Fig 2:**
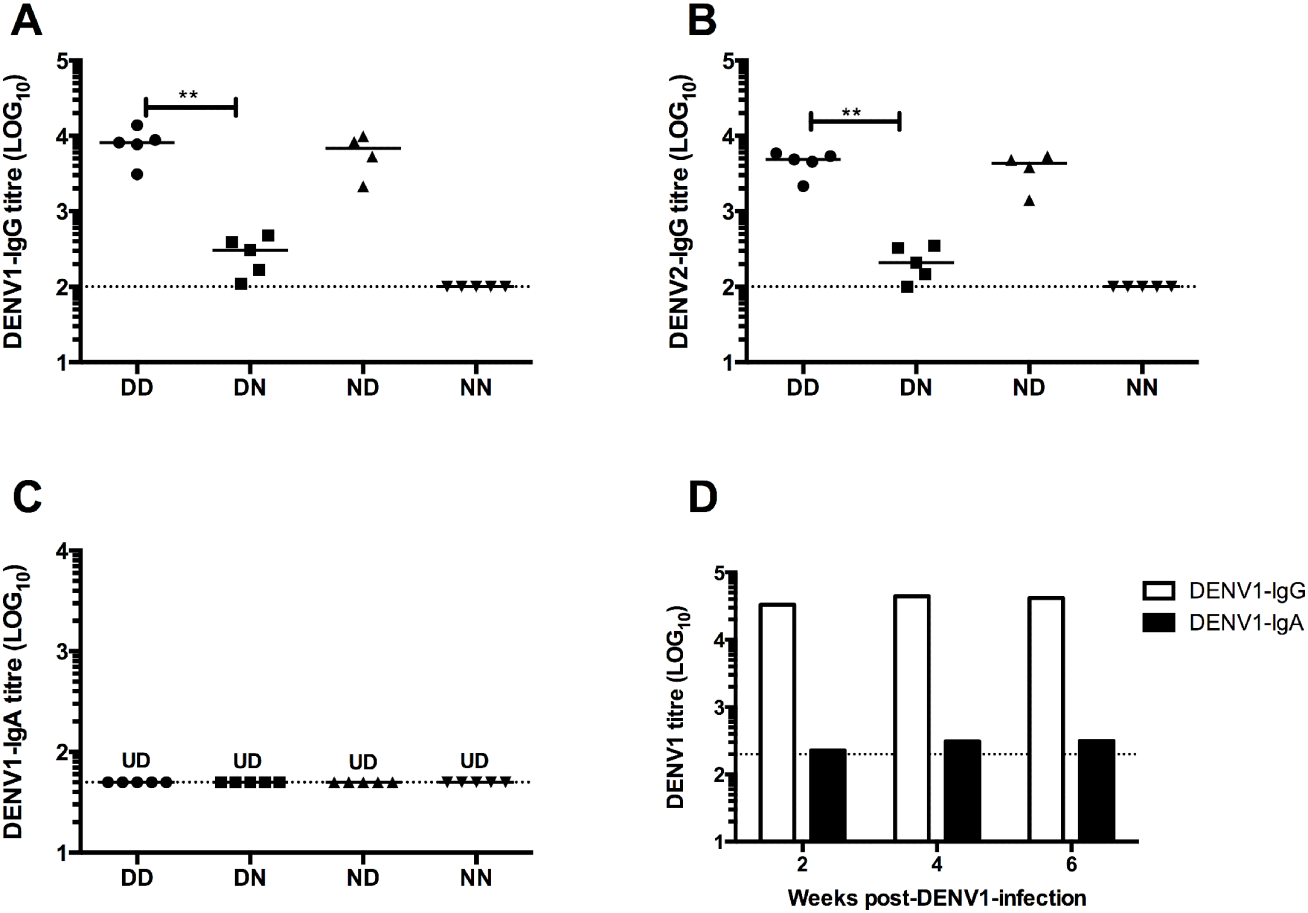
Maternally acquired dengue-specific IgG and IgA antibody titers. (A) DENV1-specific IgG and (B) cross-reactive DENV2-IgG titers in sera from 5-week old A129 mice (n = 4–6) born to and nursed by DENV1-immune (DD), or born to DENV1-immune mothers and nursed by naïve mothers (DN), or born to naïve mothers and nursed by DENV1-immune mothers (ND), or born to and nursed by naïve mothers (NN). **p<0.01 based on Mann-Whitney test comparing DN and ND with respect to DD control group. (C) DENV1-specific IgA titers in sera from 5-week old mice born to and nursed by DENV1-immune or naive mothers (n = 5). UD: Undetectable. (D) DENV1-specific IgG and IgA titers in pooled sera from mothers infected with DENV1 at week 2, 4, and 6 post-infection. Dotted line denotes the limit of detection of the assay.

Measurement of DENV1-specific IgG levels by ELISA showed that the systemic level of DENV1 IgG antibodies in DN mice (acquired maternal antibodies from gestation only) was significantly lower than that measured in DD mice (acquired antibodies from both gestation and breastfeeding) (Fig 2A). Instead, mice which acquired antibodies from breastfeeding only (ND) displayed DENV1 IgG levels that were comparable to those measured in DD control mice, suggesting that breastfeeding is the major route for maternal antibody transfer (Fig 2A). ELISA results using DENV2 as coating antigen to assess levels of cross-reactive IgG antibodies revealed similar trends (Fig 2B). Measurement of DENV1-specific IgG subclass indicated that IgG2a is the main subclass of DENV1-specific IgG acquired by pups born to and/or nursed by DENV1-immune mothers (S1 Fig). Furthermore, measurement of neutralising activity (PRNT_50_) of sera showed similar trend as that of ELISA where mice from DD and ND groups had similar neutralising titres against DENV1 and DENV2, whereas DN mice displayed lower titres (Table 1).

**Table 1:** Neutralising titres (PRNT_50_) against DENV1 and DENV2 of sera from 5-week old mice born to and/or nursed by DENV1-immune mothers. Values are expressed as mean ± standard deviation (SD) (n = 5).

PRNT_50_	DD	DN	ND	NN
**DENV1**	61.75 ± 24.21	17.73 ± 7.34	56.61 ± 17.78	<10
**DENV2**	32.74 ± 11.13	15.81 ± 4.72	26.58 ± 8.82	<10

Together, these data support that in this mouse model, DENV1-specific maternal IgG antibodies are mainly acquired from breastfeeding.

Since secretory IgA antibodies represent the main class of antibodies in breast milk [26], the level of DENV1-specific IgA antibodies was measured in the sera from mice born to and/or nursed by DENV1-immune mothers. ELISA results showed that the level of DENV1-specific IgA antibodies was below the detection limit in 5-week old mice born to and/or nursed by DENV1-immune mothers (Fig 2C). Measurement of pooled sera samples from DENV1-immune mothers also revealed levels of DENV1-specific IgA near the detection limit (Fig 2D). Thus, in this mouse model of ADE mediated by maternal antibodies, DENV1-sepcific IgA antibodies, if any, are unlikely to play a role in influencing disease outcome.

### Maternal DENV1 IgG antibodies acquired during gestation or breastfeeding enhance disease severity upon heterotypic DENV2 infection

**Fig 3:**
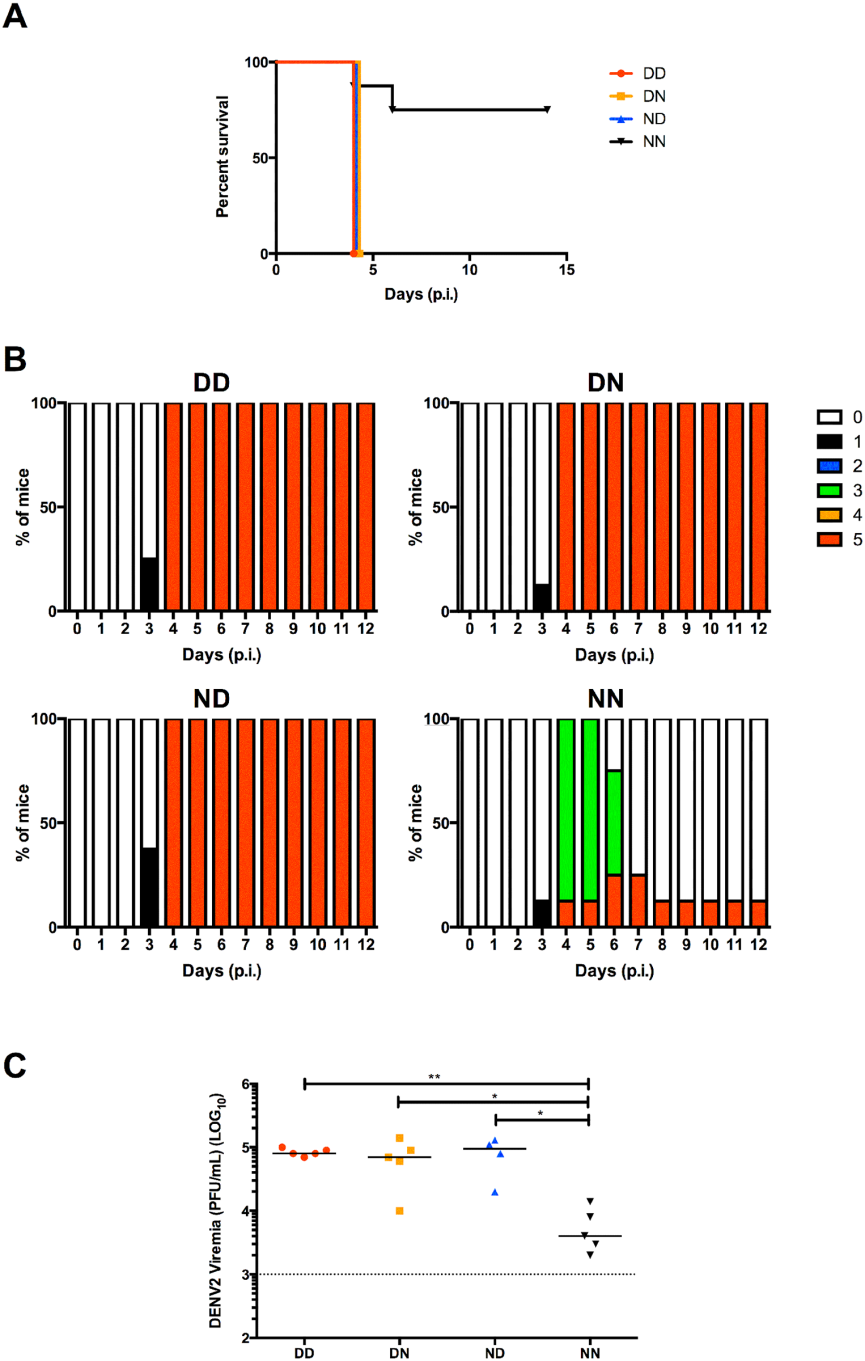
DENV2 challenge of 5-week old mice born to and/or nursed by DENV1-immune mothers. DD, DN, ND and NN groups were iv. infected with 10^6^ PFU DENV2. (A) Survival rate, (B) clinical score of individual group (0: Healthy; 1: Ruffled fur; 2: Hunched back; 3: Diarrhoea; 4: Lethargic; 5: Moribund) (n = 8) and (C) viremia measured at day 4 post-infection (n = 5). Dotted line denotes the limit of detection in plaque assay. *p<0.05; **p<0.01 based on Mann-Whitney test compared against NN control.

To assess the contribution in disease severity of maternal antibodies acquired during gestation or breastfeeding, 5-week old mice from DD, DN, ND and NN groups as described above, were challenged with a sub-lethal dose of DENV2. As previously observed [11], mice born to and nursed by DENV1-immune mothers (DD) displayed enhanced disease severity compared to control mice born to and nursed by their naïve mothers (NN) whereby DD mice were moribund by day 4 post-infection while majority of NN mice remained healthy throughout the observation period (Fig 3A and 3B). Interestingly, both ND mice (born to naïve mothers but nursed by DENV1-immune mothers) and DN mice (born to DENV1-immune mothers but nursed by naïve mothers) displayed disease kinetic and severity similar to that observed with DD mice (Fig 3A and 3B).

Furthermore, measurement of viral loads at day 4 post-infection in serum samples from DENV2-infected DD, DN and ND groups all showed increased viremia titres compared to NN controls (Fig 3C). Together, these data demonstrate the enhancing potential of maternal DENV1-IgG antibodies acquired during either gestation or breastfeeding. They also indicate that the lower level of anti-DENV1 IgG antibodies measured in DN mice (acquired during gestation only) was sufficient to enhance DENV2 infection.

### Breast milk-derived DENV1-specific IgG antibodies confer extended enhancement window

**Fig 4:**
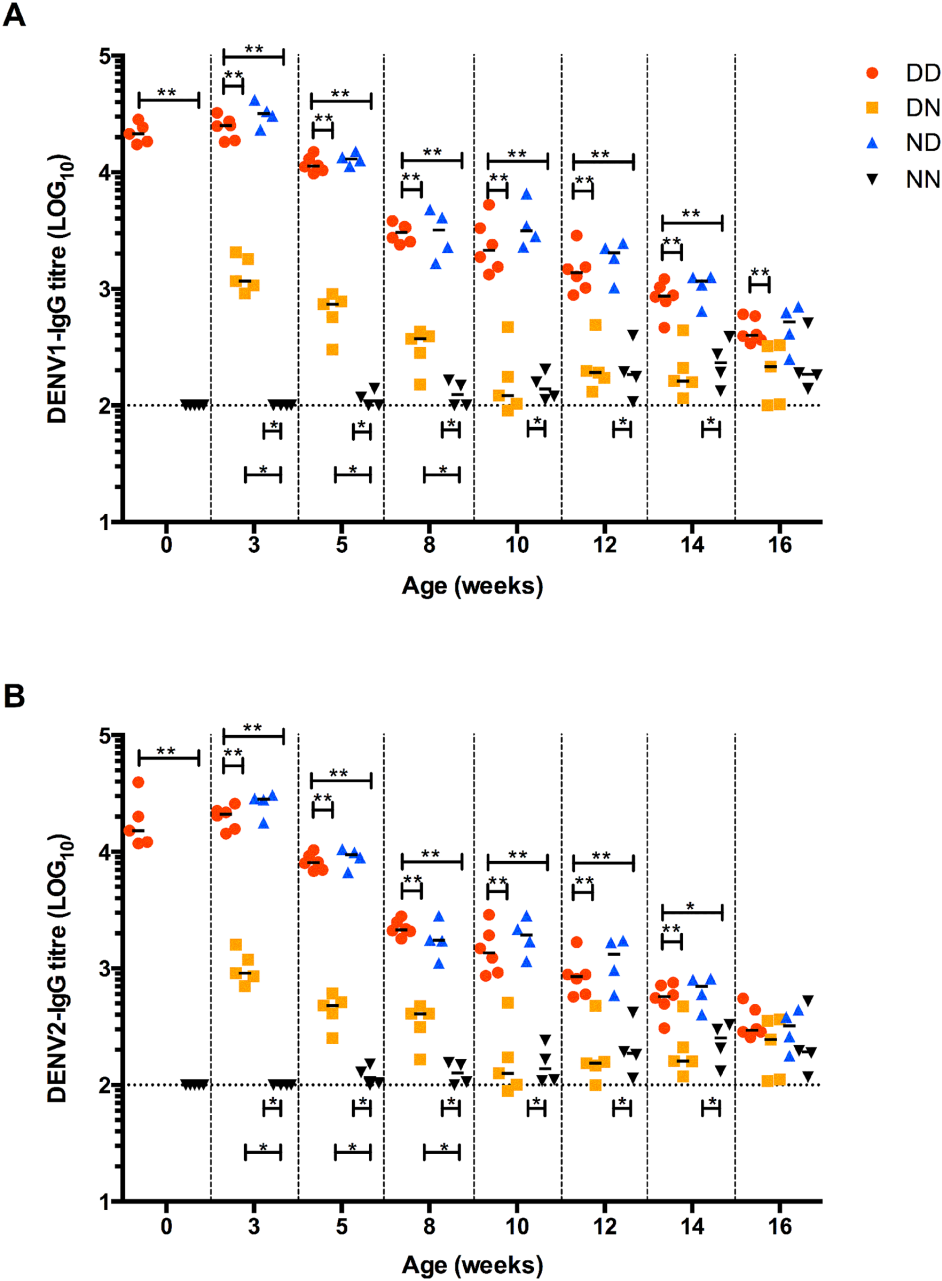
Decay of maternal DENV1-IgG antibodies over time. (A) DENV1-specific IgG and (B) cross-reactive DENV2-IgG measurement in sera from mice born to and/or nursed by DENV1-immune mothers (n = 4–5). Dotted line denotes the limit of detection of the assay. *p<0.05; **p<0.01 based on Mann-Whitney test.

Maternal antibodies in infants are catabolised with time. Dengue disease enhancement contributed by maternally acquired antibodies is thus likely to be lost over time when these antibodies decrease to non-enhancing levels [8]. Here, using the same switching strategy (Fig 1), we monitored the decay of maternal anti-DENV1 IgG antibodies acquired during gestation, breastfeeding or both. Results showed that the level of DENV1-specific IgG antibodies measured at birth in mice born to DENV1-immune mothers was comparable to the level measured in 3-week old pups born to and nursed by DENV1-immune mothers (DD) as well as in mice born to naïve dams and nursed by DENV1-immune mothers (ND) (Fig 4A). In contrast, antibody titres in 3-week old mice born to DENV1-immune dams but nursed by naïve mothers (DN) dropped by >1log (Fig 4A and Table 2). This observation thus indicated that the antibody titer measured at 3 weeks of age is mainly contributed by breastfeeding. Upon weaning at 3 weeks of age, antibody decay in all the groups was monitored over time and the antibody half-life was determined. In mice born to and/or nursed on DENV1 immune dams (DD and ND groups), the antibody titers dropped by one log between week 3 and week 8 leading to a half-life of 11.5 and 10.5 days, respectively (Fig 4A and Table 2). Between week 8 and week 16, the antibody decay in these groups was slower with antibody half-life of 16.5 and 14.5 days, respectively. These observations thus indicated a biphasic decay pattern of the maternal DENV1 IgG antibodies in pups born to and/or nursed on DENV1 immune dams. Similarly, a biphasic decay pattern was observed in mice born to DENV1 immune mothers and nursed on naïve mothers (DN group). A first phase of rapid decay between birth and 3 week of age was observed with antibody half-life of 5 days, followed by a slower decay phase between week 3 and week 10 with antibody half-life of 16.5 days (Fig 4A and Table 2). When coating the ELISA plates with DENV2 virus to study the cross-reactivity of the anti-DENV1 maternal antibodies, similar trends were observed (Fig 4B).

**Table 2:** Half-life of DENV1-IgG in DD, DN and ND groups over time.

	Birth-week 3	week 3-week 8	week 8-week 16	week 3-week 10
**DD**	-	11.5 days	16.5 days	-
**ND**	-	10.5 days	14.5 days	-
**DN**	5 days	-	-	16.5 days

Altogether, these observations thus suggested that i) majority of DENV1-specific IgG antibodies measured in 3-week old mice originates from breast milk, ii) placentally acquired dengue-specific IgG decay rapidly during the first 3 weeks after birth, and iii) decay of both placentally acquired and breast milk-derived IgG antibodies display a biphasic pattern.

**Fig 5:**
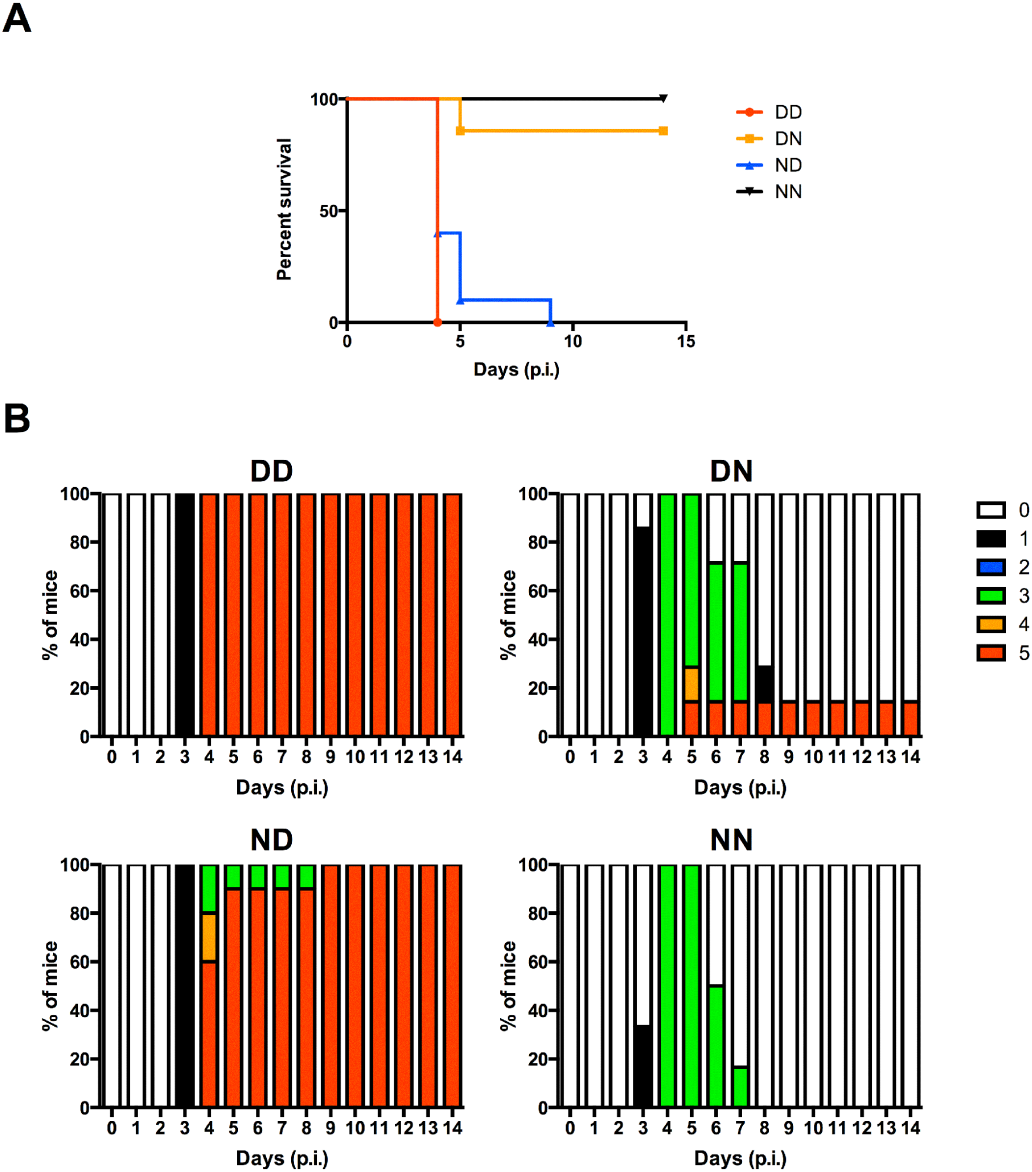
DENV2 sub-lethal challenge of 10-week old A129 mice born to and/or nursed by DENV1-immune mothers. 10-week old A129 mice from DD, DN, ND and NN groups were iv. infected with 10^6^ PFU DENV2. (A) Survival rate and (B) clinical score of individual group (0: Healthy; 1: Ruffled fur; 2: Hunched back; 3: Diarrhoea; 4: Lethargic; 5: Moribund) (n = 6–10).

Based on the ELISA results, 10-week old DN mice had negligible levels of anti-DENV1 IgG whereas 10-week old ND mice still displayed significant levels of these antibodies (Fig 4A). To test whether such difference may translate into differential disease outcomes, 10-week old mice from the different groups were challenged with a sub-lethal dose of DENV2. Mice in ND and DD groups displayed disease enhancement with majority of the mice being moribund by day 4–5 post-infection (Fig 5A). In contrast, majority of the mice from DN group survived and displayed moderate clinical manifestations that were comparable to the NN control group (Fig 5B).

Together, these data confirm that majority of DENV1-specific IgG are acquired during breastfeeding and indicate a greater enhancement window in mice nursed by DENV1-immune mothers (regardless of the immune status of their birth mothers) compared to mice born to DENV1-immune mothers but nursed by naïve mothers.

### Breastfeeding confers protection upon homologous challenge

**Fig 6:**
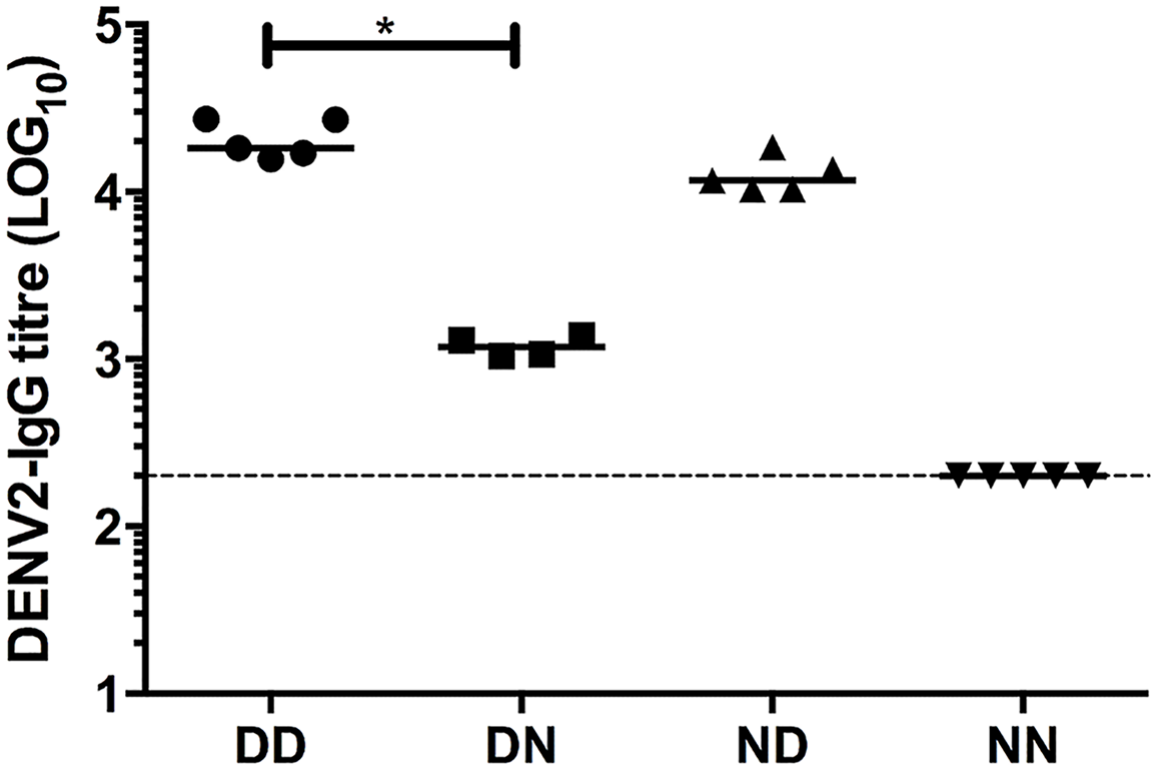
DENV2-specific IgG titres in mice born to and/or nursed by DENV2-immune mothers. DENV2-specific IgG levels were measured by ELISA in sera from 5-week old mice born to and nursed by DENV2-immune or naïve mothers (n = 4–5). Dotted line denotes the limit of detection of the assay. *p<0.05 based on Mann-Whitney test comparing DN and ND against DD control.

Besides being implicated in ADE, DENV-specific antibodies may also be involved in protection against infection. The protective role of dengue antibodies acquired from breast milk was thus examined. For this, a dengue homotypic model was set up where mice born to and nursed by DENV2-immune mothers were subsequently challenged with a lethal dose of the same DENV2 strain. The relative amounts of maternal DENV2 IgG acquired during gestation or from breastfeeding were again determined by switching pups at birth and measuring their systemic levels of DENV2 IgG at 5 weeks of age. Similar to the heterotypic ADE model, mice nursed by DENV2-immune mothers regardless of the immune status of their birth mothers (DD and ND) had comparable systemic levels of DENV2-IgG antibodies that were significantly higher than those measured in DN mice (born to DENV2-immune mothers but nursed by naïve mothers) (Fig 6). The neutralising activity (PRNT_50_) of sera against DENV2 displayed similar trends (Table 3). The comparable levels of DENV2-IgG antibodies in DD and ND groups again indicated that the main route of maternal dengue-IgG transfer is during breastfeeding.

**Table 3:** Neutralising titres (PRNT_50_) against DENV2 of sera from 5-week old mice born to and nursed by DENV2-immune or naïve mothers. Values are expressed as the mean ± SD (n = 4–5).

PRNT_50_	DD	DN	ND	NN
**DENV2**	108.09 ± 23.76	23.72 ± 4.49	74.33 ± 21.58	<10

**Fig 7:**
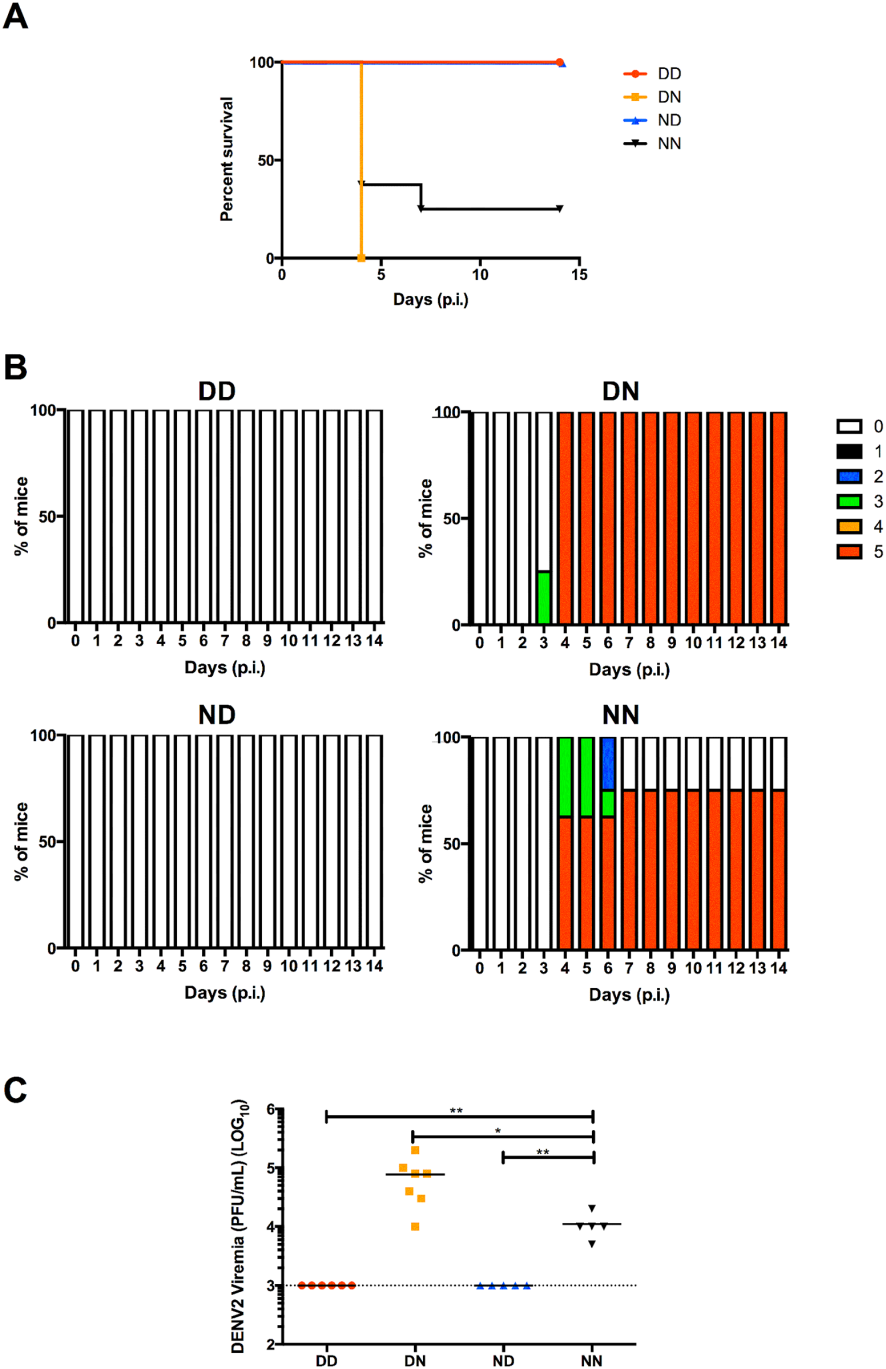
DENV2 lethal challenge of 5-week old mice born to and/or nursed by DENV2-immune mothers. 5-week old mice were infected iv. with 10^7^ PFU DENV2. (A) Survival rate, (B) clinical score of individual group (0: Healthy; 1: Ruffled fur; 2: Hunched back; 3: Diarrhoea; 4: Lethargic; 5: Moribund) (n = 8) and (C) viremia titres were measured at 4 days post-infection (n = 5–7). Dotted line denotes the limit of detection in plaque assay. *p<0.05; **p<0.01 based on Mann-Whitney test compared against NN control.

Upon lethal DENV2 challenge, all mice in DD and ND groups survived (Fig 7A) and remained asymptomatic throughout the course of the experiment (Fig 7B). These mice also had undetectable viremia levels (Fig 7C). In contrast, more than 50% of mice from NN group were moribund by day 4 post-infection (Fig 7A and 7B). Interestingly, mice in DN group displayed enhanced disease severity with 100% mortality (Fig 7A and 7B) and significantly higher virus titres compared to NN mice (Fig 7C).

Together these data suggested that 5-week old DD and ND mice, which acquired maternal DENV2-specific IgG antibodies during breastfeeding, had protective levels of maternal DENV2 antibodies. On the other hand, 5-week old DN mice, which acquired these antibodies during gestation only, had sub-neutralizing levels of maternal anti-DENV2 IgG that led to enhancement of disease severity.

## Discussion

The present study demonstrates the importance of dengue-specific IgG antibodies acquired during breastfeeding in disease enhancement and protection in a symptomatic mouse model. While maternal dengue-specific IgG antibodies acquired during either gestation, breastfeeding or via both routes enhanced dengue disease severity upon heterotypic infection, breastfeeding resulted in an extended window of disease enhancement. On the other hand, breastfeeding protected mice upon homotypic challenge, whereas the level of transplacentally acquired IgG antibodies led to enhancement of disease due to sub-neutralizing concentrations.

Human challenge studies performed by Sabin in 1952 showed long-term protection against homotypic dengue infection, but only short-term protection against heterotypic infection, after which susceptibility to disease was observed [4, 5]. Furthermore, epidemiological reports suggested increased risk of DHF/DSS development upon secondary heterotypic infection associated with pre-existing dengue antibodies [27, 28]. It was also noted that dengue disease severity is influenced by the time interval in between two sequential heterotypic infections whereby a longer time interval was associated with greater disease severity [3, 29]. In infants born to dengue immune mothers, it was observed that the risk of developing severe disease upon primary dengue infection was significantly increased between 5–9 months of age, correlating with maternal antibodies waning from protective to enhancing levels [5, 8, 18–22]. However, the relative contribution of antibodies acquired during gestation or during breastfeeding has never been investigated. Here, using our maternal antibody transfer mouse model, we found that high levels of maternal dengue-specific IgG antibodies were present in neonates at birth thus reflecting efficient IgG translocation across the placenta during gestation. However, a rapid decay of those placentally acquired IgG was observed as evidenced by >1 log drop in titres and an antibody half-life of 5 days only in 3-week old pups born to DENV1-immune dams but nursed by naïve mothers. In contrast pups born to naïve dams and breastfed for 3 weeks on DENV1-immune mothers had IgG titres that were comparable to the levels measured at birth in mice born to DENV1-immune mothers. This observation thus supported that breastfeeding provides sustained high levels of dengue-specific IgG. Interestingly, the half-life of serum DENV-IgG antibodies after 3 weeks of age ranged between 10.5 and 16.5 days, which is greater than the average half-life of 6–8 days reported for IgG antibodies in rodents [30]. This finding replicates well observations made in humans where maternal DENV-IgG antibodies persist for 24 to 150 days [31–33] whereas the average half-life of human IgG has been reported to be around 21 days [34]. Furthermore, a biphasic decay pattern was observed in all the groups characterized by an initial phase during which the maternal IgG half-life ranges from 5 to 11.5 days, followed by a second slower decay phase where the antibody half-life ranges from 14.5 to 16.5 days. Such biphasic antibody decay pattern has been previously reported in infants who maternally acquired dengue-specific IgG antibodies, and was characterized by a first decay phase with antibody half-life of 24–29 days, followed by a slower decay phase with antibody half-life of 44–150 days [31–33]. Similar observations were made in vaccination studies against pertussis and malaria, indicating that this phenomenon of biphasic antibody decay pattern is not specific to dengue [35, 36].

The importance of breastfeeding as a major maternal IgG transfer route is in line with previous literature reporting efficient intestinal FcRn-mediated IgG translocation in rodents [22, 23, 37, 38]. In humans, maternal IgG antibody transfer is believed to occur mainly through placental FcRn-mediated translocation during gestation [22–24]. However, there are very limited studies on maternal IgG antibodies in human milk and their impact on infants [21]. In addition, the role of human intestinal FcRn in facilitating IgG translocation is not widely documented. Nevertheless, the existence of FcRn in human intestinal cells has been reported [39, 40] and it was shown to be functional in bidirectional transport of IgG antibodies [41]. In addition, intestinal FcRn in human foetus was suggested to mediate maternal IgG uptake from ingested amniotic fluid during gestation [42–44]. Thus, the same intestinal FcRn may also function in postnatal IgG translocation during breastfeeding. Further investigation on human FcRn is thus necessary and may reveal a greater role and importance of breastfeeding-mediated maternal IgG transfer. Studies of breast milk antibodies indeed mainly focused on secretory IgA involved in mucosal defence [26]. However, in the context of dengue, IgA antibodies are likely to play a minimal role, as evidenced by undetectable levels of dengue-specific IgA in dengue-infected mice.

Besides maternal antibodies, immunologically active components such as lactoferrin, lysozyme and immune cells including macrophages, neutrophils and lymphocytes have also been reported to be transmitted maternally via breastfeeding [45, 46]. Transfer of maternal T- and B-lymphocytes was proposed to play a role in immune tolerance against maternal HLA [47], and to provide IgG-secreting B-lymphocytes that could possibly be involved in the maintenance of detectable levels of serum IgG in the offspring [48], respectively. However, trans-epithelial migration of maternal lymphocytes to the systemic circulation in infants remains controversial with conflicting literature, indicating that more experimental work needs to be done in order to fully assess the contribution of these maternally transferred lymphocytes [46, 49]. Nevertheless, the possibility that immunologically active components present in the breast milk from DENV1-immune mothers other than free DENV1-specific IgG antibodies may play a role in disease enhancement cannot be completely ruled out. However, the fact that passive administration of purified monoclonal enhancing IgG antibodies recapitulated ADE strongly support that IgG antibodies play an important role in triggering this phenomenon [50–52].

Current guidelines neither preclude nor encourage breastfeeding by dengue-immune mothers as this area has been unexplored [46]. Our work thus provides the first evidence of the possible role of breast milk acquired dengue-specific IgG antibodies in both dengue disease enhancement and protection in infants. However, since the four DENV serotypes co-circulate in most of the dengue endemic countries, mothers are likely to be immune to more than one serotype. In fact, majority of infants who displayed disease enhancement were found to be born from mothers immune to more than one DENV serotype [32; 53, 54]. Thus, using the same mouse model of maternal IgG transfer, it will be interesting to investigate the infection outcome in pups born to mothers immune to more than one DENV serotype. Also, a prospective study enrolling breastfeeding and non-breastfeeding dengue-immune mothers, and their babies in dengue endemic regions would provide further insight on the contribution of breast milk acquired antibodies in dengue disease and protection.

## Supporting Information

S1 Fig DENV1-specific IgG subclasses in pups born to and/or nursed by DENV1-immune mothers. ELISA of DENV1-specific IgG subclasses in the serum from DENV1-immune mothers and their 5-weeks old pups. Dotted line denotes the lowest dilution of sera and a nominal titre of 50 was assigned to samples with titre below the lowest dilution. **p<0.001 based on Mann-Whitney test.
(TIF)
